# The anatomy of the short head of biceps – not a tendon

**DOI:** 10.4103/0973-6042.63209

**Published:** 2009

**Authors:** James C.I. Crichton, Lennard Funk

**Affiliations:** The University of Manchester, UK; 1 Wrightington Hospital, UK

**Keywords:** Acromioclavicular, biceps, dissection, short head, proximal tendon

## Abstract

**Background:**

The short head of biceps brachii has been the subject of little investigation when compared to the long head or distal biceps tendons. The aim of this study was to dissect and describe the origin and proximal portion of the short head of biceps brachii.

**Materials and Methods:**

Three left and two right (*n* = 5) fresh-frozen human cadaver shoulders were dissected and the proximal short head was measured and photographed.

**Results:**

The origin of the short head of biceps consisted of muscle fibres attaching directly to the tip of the coracoid process, with a thin, tendinous aponeurosis covering its anterior surface, rather than a true tendon as previously described.

**Conclusion:**

The short head of biceps does not attach to the coracoid process via a true tendon. These findings have implications for procedures that utilise the short head of biceps.

**Level of Evidence:**

Basic science study.

## INTRODUCTION

The tendon of the short head of biceps brachii has been the subject of little investigation when compared to the long head or distal biceps tendons. Much is known about the macroscopic and microscopic anatomy, physiology and pathology of the latter two tendons[1–5] but few papers deal specifically with the short head and these seldom include detailed description of the anatomy of the proximal attachment.

The relative infrequency of injuries involving the short head[6] may explain why it is a less popular topic for researchers. The short head has, however, an important role in certain acromioclavicular (AC) joint reconstruction procedures. The authors utilise the short head of biceps to maintain reduction of a dislocated clavicle in a modification of the procedure described by Vargas.[7] We utilise 1 or 2 centimetres of the superficial ‘tendon’, which is then reflected upwards, threaded through a hole made in the clavicle and then sutured to itself. Whilst conducting this procedure the authors observed that the proximal short head does not appear to be, as described in anatomical texts, a true tendon.

Understanding the tendon's features is useful in developing and refining procedures similar to that utilised by the authors to reconstruct the dislocated acromioclavicular joint. The aim of this study was to dissect and describe the origin and proximal portion of the short head of biceps brachii.

## MATERIALS AND METHODS

Three left and two right (*n*=5) fresh-frozen human cadaver shoulders were utilised, from separate cadavers.

The coracoid and short head of biceps were carefully dissected and identified. The intact short head of biceps was measured using Vernier callipers. Length of the tendinous portion was measured from the tip of the coracoid process to the termination of visible tendinous fibres blending into the muscle belly. Width and thickness were measured 20 mm distal to the tip of the coracoid process with the muscle flattened as much as possible. Cross-sectional area was calculated as width multiplied by thickness.

The short head was then cut as close as possible to the coracoid process. The free muscle was reflected inferiorly to allow examination of its attachment, cross-sectional features and posterior surface.

Particular attention was paid to the characteristics and composition of the proximal muscle, with a view to asserting whether the attachment was via a tendon or a direct muscular attachment. Measurements of the proximal muscle were taken to provide quantitative morphological data and comparison between specimens.

## RESULTS

**Figure 1:**
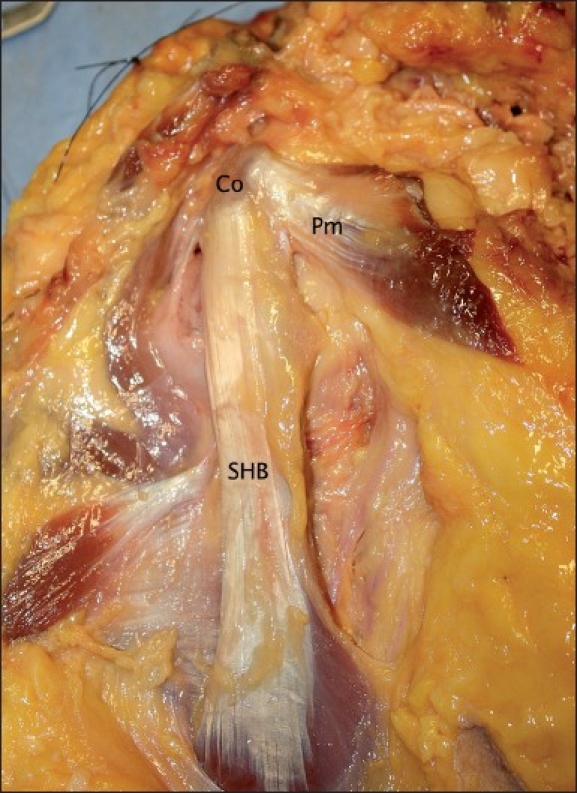
The proximal short head of biceps (Co = Coracoid process; Pm = Pectoralis minor muscle; SHB = Short head of biceps muscle)

The origin of the short head of biceps was seen to consist of muscle fibres attaching directly to the tip of the coracoid process with a thick, tendinous aponeurosis covering the anterior surface of the muscle for a distance of at least 98mm (range 98 – 123 mm). Viewing the intact short head anteriorly, the fibres of the aponeurosis could be seen to insert into the muscle belly [Figure 1].

**Figure 2:**
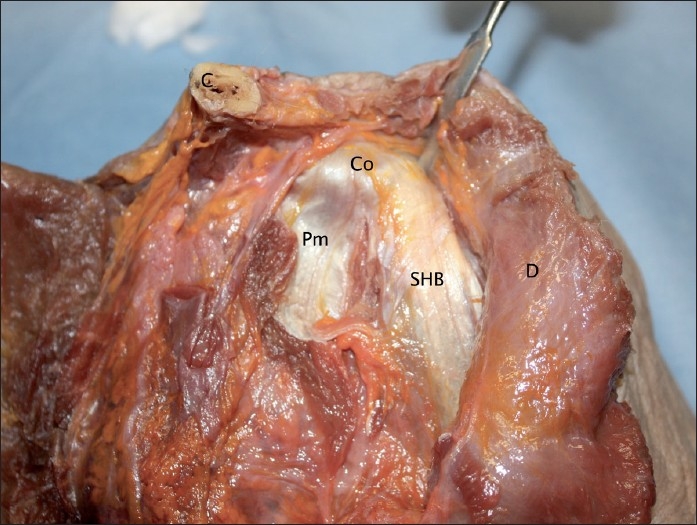
The coracoid process with attached short head and pectoralis minor, specimen 1 (Co = Coracoid process; Pm = Pectoralis minor muscle; SHB = Short head of biceps muscle; C = Clavicle; D = Deltoid muscle)

**Figure 3:**
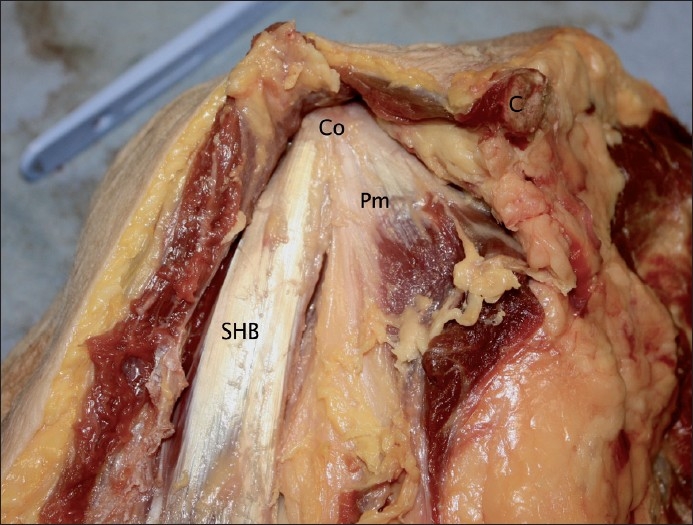
The coracoid process with attached short head and pectoralis minor, specimen 5 (Co = Coracoid process; Pm = Pectoralis minor muscle; SHB = Short head of biceps muscle; C = Clavicle)

In all specimens the fibres of the tendinous aponeurosis and muscle itself descended vertically from their attachment at the apex of the coracoid process. This attachment did not differ between specimens. The aponeurosis completely covered the tip of the coracoid process and the proximal muscle fibres. The tendon of pectoralis minor attached to the coracoid adjacent and medial to the short head, but fibres of this tendon were separate from fibres forming the short head. Figures 2 and 3 show the short head and pectoralis minor at the coracoid process in two different specimens.

**Figure 4:**
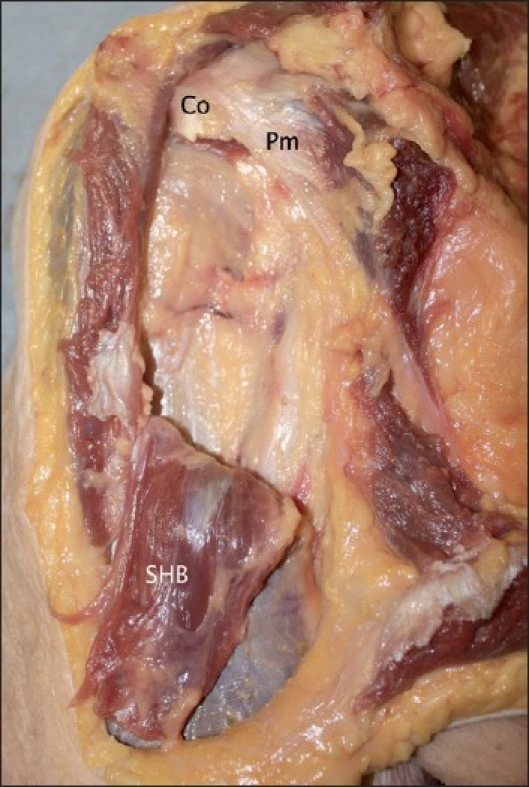
The short head of biceps, transected and reflected inferiorly (Co = Coracoid process; Pm = Pectoralis minor muscle; SHB = Short head of biceps muscle)

Transecting the short head off the coracoid process demonstrated the characteristics of its attachment. The short head of biceps attachment constituted a direct muscular attachment to the coracoid process with a thick aponeurosis covering these muscle fibres anteriorly in all specimens. The epimysium of the muscle blends with the periosteum of the bone without an intermediary tendon in a direct attachment. Dividing the short head and reflecting it inferiorly highlights this as seen in Figure 4. When reflected and viewed from the underside, the short head is clearly muscular from its origin.

**Figure 5:**
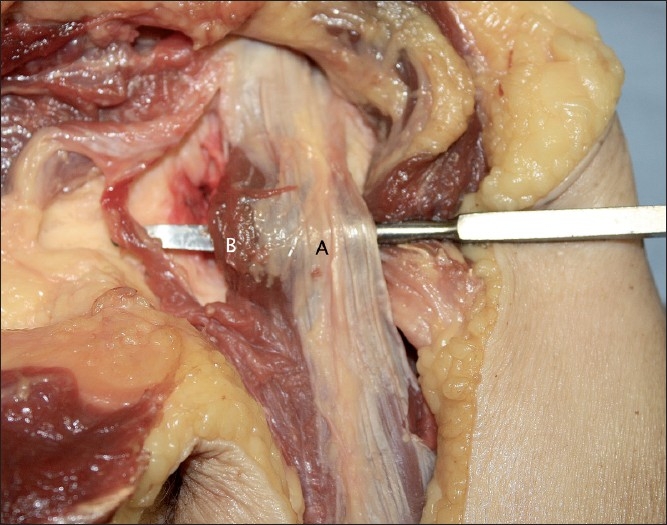
The aponeurosis covering the short head, with a bare area medially (A = Aponeurosis; B = Bare area of short head)

**Figure 6:**
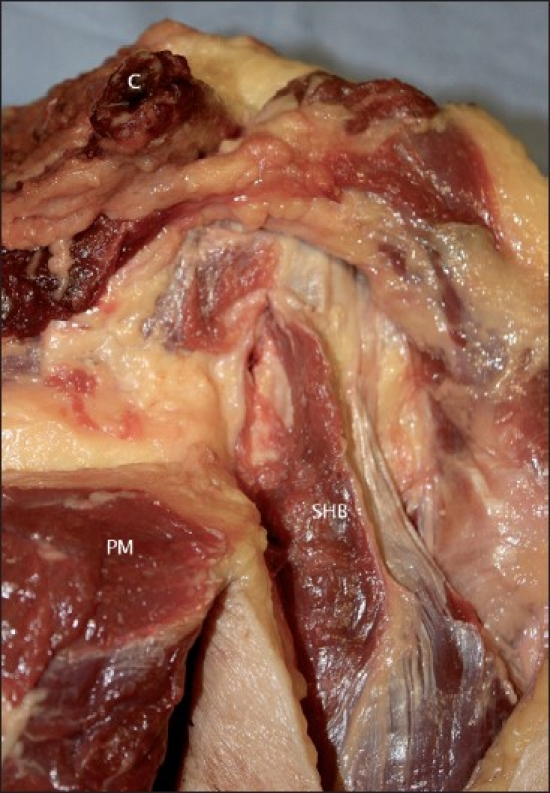
The short head rotated 90 degrees to show its underside (C = Clavicle; PM = Pectoralis major muscle; SHB = Short head of biceps muscle)

Medially, the muscle fibres were not covered by this aponeurosis from just distal to their origin. This was the case in all specimens. These fibres are likely to contribute predominantly to the coracobrachialis. It was not, however, possible to make a distinction between fibres of this muscle and those of the short head. The attachment of muscular fibres to the coracoid did not differ from medial to lateral. The aponeurosis and medial bare area are labelled in Figure 5. Figure 6 shows the intact short head rotated 90 degrees about its longitudinal axis to show the anterior aponeurosis and underlying muscle.

**Figure 7:**
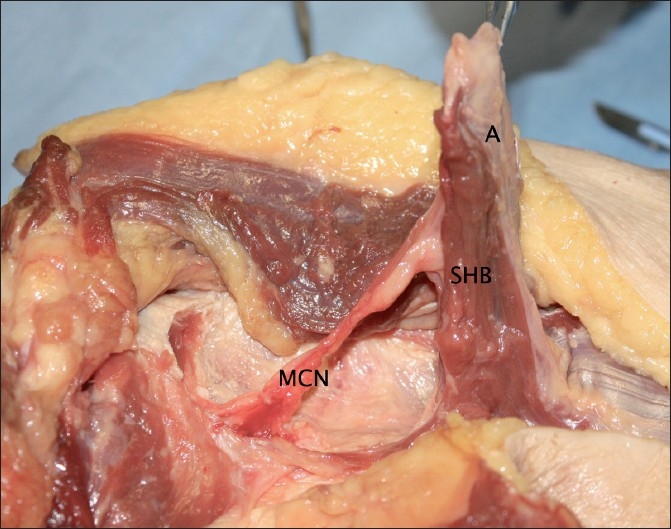
The relationship of the musculocutaneous nerve to the short head (A = Aponeurosis; SHB = Short head of biceps muscle; MCN = Musculocutaneous nerve)

The musculocutaneous nerve was identified in all specimens, passing inferolaterally posterior to the short head. It can be seen in the Figure 7, which also shows the relationship of the aponeurosis and the muscle belly.

**Figure 8:**
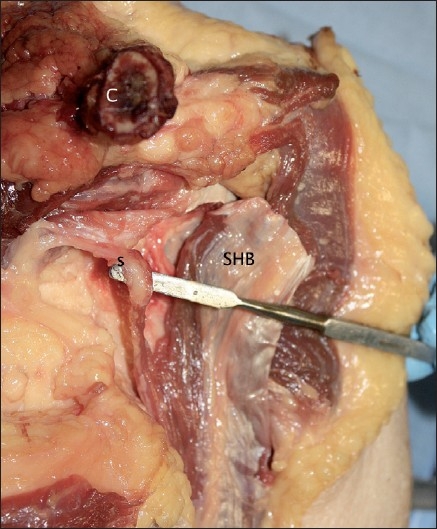
A small muscular slip contributing to the short head (SHB = Short head of biceps muscle; C = Clavicle; S = Muscle slip)

The cross-sectional profile of the short head was rectangular. The aponeurosis made up approximately one-quarter of its thickness. In specimen 4, a small muscular slip was found, which originated from the fascia overlying the pectoralis minor. This blended with the rest of the muscular fibres approximately 50 mm distal to the coracoid process. This was not seen in any other specimens. Figure 8 shows this muscular slip.

The measurements taken from each tendon are described in Table 1. The aponeurosis in specimens was approximately the same length with the exception of specimen 5, which was considerably shorter. The width of specimen 5 was also less than other specimens. The mean and standard deviation were calculated for each set of results.

**Table 1:** Measurements of the proximal short head of biceps and aponeurosi

Specimen (mm)	Length (mm)	Width (mm)	Thickness (mm)	Cross-sectional Area (mm^2^)
1	122.0	24.0	4.0	96.0
2	122.0	24.0	6.0	144.0
3	120.0	30.0	7.0	210.0
4	123.0	30.0	6.0	180.0
5	98.0	13.5	6.0	81.0
Mean	117	24.3	5.8	142.2
Standard deviation	10.67	6.74	1.1	54.6

A Shapiro-Wilk W test for non-normality was carried out on the Length, Width and Thickness groups of data, using StatsDirect 2006 statistical software. The data for Length are unlikely to be from a normal distribution (*W* = 0.641153, *P* = 0.0021). Data for Width (*W* = 0.847772, *P* = 0.1876) and Thickness (*W* = 0.828273, *P* = 0.135) show no evidence of non-normality.

## DISCUSSION

The results suggest that the attachment of the short head of biceps to the coracoid is a muscular attachment with an aponeurosis on its anterior surface. This was seen in all five specimens with little variation. This is in contrast to previous descriptions of the attachment. In the literature, descriptions of the anatomy of the short head are sparse. Studies specifically concerning the short head have taken the form of case reports of traumatic rupture,[6,8] with the anatomical features of the region being mentioned only briefly. Most texts report a short, flat tendon between the origin at the coracoid process and the muscle belly itself.[9] Doyle *et al.* describe a thick, flat tendon arising from the tip of coracoid process that is conjoint with the tendon of coracobrachialis. This separates and the muscle fibres descend vertically from the dorsomedial surface of the tendon.[10] Moore, Dalley and Agur simply describe a tendinous attachment to the coracoid process of the scapula.[11]

Aponeurosis is defined as a ribbon-like tendinous expansion, whereas tendons are defined as fibrous cords of connective tissue.[12] Of the five specimens we studied, all had a reasonably long region that resembled a flattened tendon, until dissected further to reveal it was in fact a thin aponeurosis covering the anterior surface of the short head of biceps. Of the papers that describe the anatomy of the short head, one describes a medial muscular side with a more tendinous lateral half.[13] This did not appear to be the case in our specimens where the distribution of muscular and overlying aponeurosis fibres appears fairly uniform in the transverse plane.

Direct muscular attachments can be seen at several sites in the body. Perhaps the most similar to the attachment we found is that of the triceps brachii at its insertion. Though this is a distal attachment, rather than the proximal attachment of the short head of biceps, it is comparable, consisting of a thick muscular attachment directly to the olecranon process with a thin aponeurosis covering its median third. The significance of this type of attachment is not apparent. Indeed, it is markedly different to the origin of the long head and insertion of the distal biceps, both of which consist of distinct tendons that attach the muscle belly to bone and, in the case of the long head, the glenoid labrum.

Some variation was noted in two of the specimens. Both specimens 3 and 4 had a slight thickening of the aponeurosis in the lateral third of the muscle. This did not appear to be a discrete tendon. These specimens appeared to have greater hypertrophy of the short head and other surrounding muscles, which may have caused this thickening of the aponeurosis. It is possible that a thickening exists in all specimens, but was only pronounced enough to be felt in the more muscular examples. Further investigation, including histology, would be necessary to demonstrate whether this is the case. It is conceivable that this is what Jiang *et al*.[14] are referring to when they describe a tendinous lateral half of the short head tendon.

In specimen 4, a small, muscular slip contributing to the short head but with a different origin was discovered. This appeared to arise from fascia overlying the pectoralis minor. No similar structure was seen in the other specimens. Specimen 5 was narrower and shorter than other specimens, although its thickness was comparable to the others. This specimen appeared to be generally smaller with less muscle bulk than the other specimens, which may account for the smaller size of the muscle. Unfortunately, we did not have the anthropomorphic data for our specimens, but it is likely that specimen 5 came from a smaller and more petite individual.

The Shapiro-Wilk test suggests that data for width and thickness are likely to have come from normal distributions. The test suggests, however, that data for length is not from a normal distribution. A glance at the data easily locates one result that differs markedly from the rest; that is, the length measurement for specimen 5. This is most likely a normal measurement at the lower end of the normal distribution. The small sample size reduces the power of the Shapiro-Wilk test, though it is regarded as the most reliable method for ascertaining evidence of non-normality in small samples.[15] A larger sample and statistical analysis would demonstrate with more certainty whether this data does come from a normal distribution and whether Specimen 5's measurements are anomalous.

Both the coracobrachialis and the short head of biceps are known to originate from the coracoid process, the coracobrachialis being more medial.[8] This proximal attachment of the two muscles is often referred to as a conjoined tendon.[13] The results of this dissection suggest that in fact both muscles attach directly to the coracoid and the two could not be distinguished at their attachment. The coracobrachialis has been recognised previously to be muscular from near its origin; Sloan *et al.*[13] describe a more muscular medial side, the coracobrachialis, and a tendinous lateral portion. The direct attachment, however, appeared to be consistently muscular from medial to lateral in all the specimens. Furthermore, the fibres of coracobrachialis and the short head of biceps were indistinguishable proximally, blending together before their shared attachment in similar fashion to the distal merging of the long and short heads of biceps.

Knowledge of the short head's attachment is useful for surgeons planning procedures to incorporate this in repairs of dislocations of the AC joint. Two main techniques have been developed, broadly classified as either static or dynamic. Use of the short head tendon for repairing AC joint dislocation was first described by Vargas in 1942,[7] though the author credits the invention of the technique to a colleague, De Carvalho. The short head is split longitudinally and then incised just proximal to the muscle fibres. This section, still attached proximally to the coracoid process, is reflected superiorly and passed through a hole made in the distal clavicle before being sutured to itself. A variation of this procedure involves attaching the reflected lateral half of the short head to the distal end of the clavicle, which has been resected, reducing its length by around 7-8mm.[14] The senior author has capitalised on the fact that the short head does not comprise a true tendon and utilises only part of the thick aponeurosis, leaving the direct muscular attachments on the coracoid.

The tensile strength of the short head is comparable to that of the CA ligament[9] and a much longer and thicker graft can be harvested than the CA ligament can provide. It is particularly beneficial in cases of failed Weaver-Dunn reconstructions. Previous studies utilising the short head of biceps have shown it to be an effective graft source, with excellent clinical and radiological outcomes.[7,13,14]

The main limitation of this study is the small sample size. The characteristics of the attachment were demonstrated consistently between the specimens, so it is not unreasonable to conclude that this is representative of the wider population. It is, however, insufficient to provide evidence of anatomical variations. Further investigation with a greater sample size is warranted if anatomical variations are to be elucidated. The other limitation of this study is the lack of information on the gender and age of the specimens. This would have helped clarify the smaller size of sample 5. However, all the other specimens were very similar.

## CONCLUSION

The short head of biceps does not attach to the coracoid process via a true tendon. Instead, the muscle attaches directly to the bone with a thin aponeurosis covering it anteriorly. Knowledge of the attachment characteristics of the short head of biceps is essential for its assessment and use in AC joint repair procedures.
